# Heritable genomic diversity in breast cancer driver genes and associations with risk in a Chilean population

**DOI:** 10.1186/s40659-022-00384-4

**Published:** 2022-05-31

**Authors:** Sebastian Morales-Pison, Patricio Gonzalez-Hormazabal, Julio C. Tapia, Alexis Salas-Burgos, Sandra Ampuero, Fernando Gómez, Enrique Waugh, José Miguel Reyes, Lilian Jara

**Affiliations:** 1 Programa de Genética Humana, Instituto de Ciencia Biomédicas (ICBM), Facultad de Medicina, Universidad de Chile, 8380453 Santiago, Chile; 2 Laboratorio de Transformación Celular, Programa de Biología Celular y Molecular, Facultad de Medicina, Universidad de Chile, 8380453 Santiago, Chile; 3 Departamento of Farmacología, Universidad de Concepción, 4030000 Concepción, Chile; 4 Programa de Virología, Instituto de Ciencias Biomédicas, Facultad de Medicina, Universidad de Chile, 8380453 Santiago, Chile; 5 Clínica Santa María, 7520378 Santiago, Chile; 6 Clínica Las Condes, 7591047 Santiago, Chile

**Keywords:** Breast cancer susceptibility genes, Driver genes, Germline variations, Single nucleotide polymorphisms, Protein function

## Abstract

**Background:**

Driver mutations are the genetic components responsible for tumor initiation and progression. These variants, which may be inherited, influence cancer risk and therefore underlie many familial cancers. The present study examines the potential association between SNPs in driver genes *SF3B1* (rs4685), *TBX3* (rs12366395, rs8853, and rs1061651) and *MAP3K1* (rs72758040) and BC in *BRCA1/2*-negative Chilean families.

**Methods:**

The SNPs were genotyped in 486 BC cases and 1258 controls by TaqMan Assay.

**Results:**

Our data do not support an association between rs4685:C > T, rs8853:T > C, or rs1061651:T > C and BC risk. However, the rs12366395-G allele (A/G + G/G) was associated with risk in families with a strong history of BC (OR = 1.2 [95% CI 1.0–1.6] p = 0.02 and OR = 1.5 [95% CI 1.0–2.2] *p* = 0.02, respectively). Moreover, rs72758040-C was associated with increased risk in cases with a moderate-to-strong family history of BC (OR = 1.3 [95% CI 1.0–1.7] *p* = 0.02 and OR = 1.3 [95% CI 1.0–1.8] *p* = 0.03 respectively). Finally, risk was significantly higher in homozygous C/C cases from families with a moderate-to-strong BC history (OR = 1.8 [95% CI 1.0–3.1] *p* = 0.03 and OR = 1.9 [95% CI 1.1–3.4] *p* = 0.01, respectively). We also evaluated the combined impact of rs12366395-G and rs72758040-C. Familial BC risk increased in a dose-dependent manner with risk allele count, reflecting an additive effect (*p*-trend = 0.0002).

**Conclusions:**

Our study suggests that germline variants in driver genes *TBX3* (rs12366395) and *MAP3K1* (rs72758040) may influence BC risk in *BRCA1/2*-negative Chilean families. Moreover, the presence of rs12366395-G and rs72758040-C could increase BC risk in a Chilean population.

## Background

Breast cancer (BC) is the preponderant malignancy among women worldwide and exacts the highest annual toll of cancer deaths in Chile (15.7/100,000 women). Unfortunately, national incidence is climbing in both younger and older populations [1]. Risk factors include gender, age, hormonal factors, and, most significantly, family history. Although genetic predisposition plays an important role in the etiology of this disease, the major susceptibility genes *BRCA1* and *BRCA2* only account for about 16% of diagnoses. Moderate- and low-penetrance genes are likely responsible for a significant percentage of familial BC in *BRCA1/2*-negative families. Many new hereditary BC (HBC) susceptibility genes were discovered between 2012 and 2015. However, all known BC susceptibility genes account for about half of hereditary *BRCA1/2*-negative BC cases, leaving much of the genetic risk unexplained.

Cancer is fundamentally a genomic disease. While numerous somatic mutations accumulate during tumorigenesis, the majority of these variants are neutral “passenger” mutations. Those variations that do contribute to tumorigenesis are known as “driver” mutations [2]. A tumor typically contains 2–8 driver mutations that initiate carcinogenesis [3–5]. The driver mutations and mutational processes operative in BC have not yet been comprehensively defined [6].

Several studies have used next-generation sequencing (NGS) to identify potential driver mutations [6–9]. Various relevant genes have been identified in sporadic breast tumors: *ARID1B*, *CASP8*, *MAP3K1, MAP3K13, NCOR1, SAMRCD1, CDKN1B, AKT2*, and *TBX3*. However, there has been scant research into the possibility that these driver genes contain inherited variants that influence the development of cancer [10]. In one of these few studies, Göhler et al. [10] investigated whether known driver genes contain heritable variants that influence risk and/or survival in Swedish BC patients. That group evaluated selected single-nucleotide polymorphisms (SNPs) located in 15 genes that have consistently been classified as BC driver genes by NGS. Five genes were associated with BC risk: *TBX3* (rs2242442) was associated with decreased risk; *TTN* (10497520) and *MAP3K1* (rs702688 and rs72758040) were associated with increased risk; *MLL2* (rs11168827) was associated with increased overall risk, positive hormone receptor status, and low-grade tumors; and *SF3B1* (rs4688) had a protective effect and was associated with negative lymph node findings, metastasis, and hormone receptor status [11]. Considering that variations in these novel driver genes had not been assessed in any Latin American population, our group performed an association study on germline variations in BC driver genes in a Chilean population. We evaluated associations between SNPs in the driver genes *TTN* (rs10497520), *TBX3* (rs2242442), *MLL2* (rs11168827), and *MAP3K1* (rs702688 and rs702689) with BC risk in *BRCA1/2*-negative Chilean families. The results did not support an association between rs702688:A > G (*MAP3K1*) or rs702689:G > A (*MAP3K1*) and risk. The rs10497520 (*TTN*) T allele was associated with decreased risk in patients with a family history of BC or early-onset BC (OR = 0.6, p < 0.0001 and OR = 0.7, p = 0.05, respectively), and rs2242442-G (*TBX3*) also demonstrated a protective effect (OR = 0.6, p = 0.02). On the other hand, rs11168827-C (*MLL2*) was linked to increased risk in families with a strong history of BC (OR = 1.4, p = 0.05) [12].

The *SF3B1* gene encodes subunit 1 of the splicing factor 3b protein complex. Several studies have identified *SF3B1* mutations in solid tumors, including 9.7% of uveal melanomas, 4% of pancreatic cancers, and 1.8% of BC [13]. The T-box 3 gene (*TBX3*) is a member of the T-box gene family. Functional analysis has shown that T-box family members are transcription factors with a highly-conserved DNA binding domain known as the T-box and a nuclear localization signal. These proteins can activate and/or repress target genes by binding to T-elements [14]. *TBX3* is a critical developmental regulator of several structures but has no known function in adult tissue. Nevertheless, *TBX3* is frequently overexpressed in several cancers, such as colon cancer, hepatocarcinoma, melanoma, chondrosarcoma, and BC. The identification of *TBX3* mutations in breast tumors samples suggests that *TBX3* is a driver gene in BC [15]. The protein *MAP3K1*, on the other hand, acts within the MAP-signaling pathway, which triggers expression of genes important for angiogenesis, proliferation, and cell migration [6]. Moreover, there is evidence to suggest that *MAP3K1* is a potential driver gene in BC [6]. On the other hand, post-translational modifications of TBX3 include phosphorylation of 29 sites in which some MAP kinase proteins are involved. Therefore, it is very important to determine whether inherited genetic variants in *SF3B1*, *TBX3*, and *MAP3K1* genes affect BC risk.

The present study evaluates the association between specific SNPs and SNP-SNP interactions in the driver genes *SF3B1*, *TBX3*, and *MAP3K1* with familial and early-onset sporadic BC, studying cases and controls from Chilean families who are negative for *BRCA1/2* point mutations. A case–control design was used to explore the relationship between BC susceptibility and the following SNPs: rs4685 (*SF3B1*), rs12366395 (*TBX3*), rs72758040 (*MAP3K1*), rs8853 (*TBX3*), and rs1061651 (*TBX3*). Moreover, we assessed the SNP-SNP interaction for rs12366395 and rs72758040 to evaluate their combined effect on BC risk. The SNPs selected in this study were chosen based on their genetic location and their possible consequence within the gene. In addition, it is important to replicate the previous association studies of these SNPs in other populations in order to confirm their effect on BC risk.

## Results

### Association between rs4685, rs12366395, rs72758040, rs8853, and rs1061651 SNPs and familial or early-onset sporadic breast cancer in non-carriers of BRCA1/2 mutations

The whole case sample was subdivided into two groups: cases with two or more family members with BC and/or OC (n = 308) (subgroup A) and non-familial early-onset BC (diagnosis at ≤ 50 years of age) (n = 178) (subgroup B). Table 1 shows the genotype and allele frequencies of the rs4685:C > T (*SF3B1*), rs12366395:A > G (*TBX3*), rs72758040:G > C (*MAP3K1*), rs8853:C > T (*TBX3*), and rs1061651:T > C (*TBX3*) polymorphisms in the whole data set, subgroups A and B, and controls. The observed genotype frequencies were in Hardy–Weinberg equilibrium for four of the five polymorphisms in controls (rs4685:C > T, p = 0.06; rs12366395:A > G, p = 0.834; rs8853:T > C, p = 0.164; rs1061651:T > C, p = 0.122), while the *p*-value was < 0.0001 for rs72758040:G > C.

**Table 1:** Genotype and allele frequencies of rs4685 (*SF3B1)*, rs12366395 (TBX3), rs72758040 (*MAP3K1)*, rs8853 *(TBX3)*, and rs1061651 (*TBX3*) in *BRCA1/2*-negative breast cancer cases and controls

		All BC cases (n = 486)			Families with ≥ 2 BC and/or OC cases (n = 308)			Families with a single case, diagnosis at ≤ 50 years of age (n = 178)		
Genotype or allele	Controls (%) (n = 1258)	BC cases (%)	OR [95% CI]	*p*-value^a^	BC cases (%)	OR [95% CI]	*p*-value^a^	BC cases (%)	OR [95% CI]	*p*-value^a^
rs4685 (*SF3B1)*										
C/C	504 (40.1)	213 (43.8)	(ref.)	–	133 (43.2)	(ref.)	–	80 (44.9)	(ref.)	–
C/T	560 (44.5)	213 (43.8)	0.9 [0.7–1.1]	0.35	139 (45.1)	0.9 [0.7–1.2]	0.68	74 (41.6)	0.8 [0.5–1.1]	0.30
T/T	194 (15.4)	60 (12.4)	0.7 [0.5–1.0]	0.07	36 (11.7)	0.7 [0.4–1.0]	0.09	24 (13.5)	0.7 [0.4–1.2]	0.34
C/T + T/T	754 (59.9)	273 (56.2)	0.8 [0.6–1.0]	0.15	175 (56.8)	0.8 [0.6–1.1]	0.33	98 (55.1)	0.8 [0.5–1.1]	0.22
Allele C	1568 (62.3)	639 (65.7)	(ref.)	–	405 (65.7)	(ref.)	–	234 (65.7)	(ref.)	–
Allele T	948 (37.7)	333 (34.3)	0.8 [0.7–1.0]	0.06	211 (34.3)	0.8 [0.7–1.0]	0.12	122 (34.3)	0.8 [0.6–1.0]	0.23
rs12366395 (*TBX3*)										
A/A	989 (78.6)	355 (73.0)	(ref.)	–	220 (71.4)	(ref.)	–	135 (75.8)	(ref.)	–
A/G	252 (20.0)	**124 (25.5)**	**1.3 [1.0–1.7]**	**0.01**	**82 (26.6)**	**1.4 [1.0–1.9]**	**0.01**	42 (23.6)	1.2 [0.8–1.7]	0.3
G/G	17 (1.4)	7 (1.4)	1.1 [0.4–2.7]	0.8	6 (1.9)	1.5 [0.6–4.0]	0.4	1 (0.6)	0.4 [0.05–3.2]	0.7
A/G + G/G	269 (21.4)	**131 (27.0)**	**1.3 [1.0–1.7]**	**0.01**	**88 (28.6)**	**1.4 [1.1–1.9]**	**0.008**	43 (24.2)	1.1 [0.8–1.6]	0.4
Allele A	2230 (88.6)	834 (85.8)	(ref.)	–	522 (84.7)	(ref.)	–	312 (87.6)	(ref.)	–
Allele G	286(11.4)	**138 (14.2)**	**1.2 [1.0–1.6]**	**0.02**	**94 (15.3)**	**1.4 [1.0–1.8]**	**0.009**	44 (12.4)	1.1 [0.7–1.5]	0.6
rs72758040 (*MAP3K1)*										
G/G	866 (68.8)	317 (65.4)	(ref.)	–	194 (63.3)	(ref.)	–	123 (69.1)	(ref.)	–
G/C	307 (24.4)	119 (24.5)	1.0 [0.8–1.3]	0.65	76 (24.7)	1.1 [0.8–1.4]	0.54	43 (24.2)	0.9 [0.6–1.4]	1.0
C/C	85 (6.8)	**50 (10.1)**	**1.6 [1.1–2.3]**	**0.01**	**38 (12.0)**	**1.9 [1.3–3.0]**	**0.001**	12 (6.7)	0.9 [0.5–1.8]	1.0
G/C + C/C	392 (31.2)	168 (34.6)	1.1 [0.9–1.4]	0.16	113 (36.7)	1.2 [0.9–1.6]	0.06	55 (30.9)	0.9 [0.7–1.3]	1.0
Allele G	2039 (81.0)	755 (77.7)	(ref.)	–	466 (75.6)	(ref.)	-	289 (81.2)	(ref.)	–
Allele C	477 (19.0)	**217 (22.3)**	**1.2 [1.0–1.4]**	**0.02**	**150 (24.4)**	**1.3 [1.1–1.6]**	**0.003**	67 (18.8)	0.9 [0.7–1.3]	0.9
rs8853 *(TBX3)*										
T/T	512 (40.7)	195 (40.1)	(ref.)	–	125 (40.6)	(ref.)	–	70 (39.3)	(ref.)	–
T/C	563 (44.8)	217 (44.7)	1.0 [0.8–1.2]	0.95	132 (42.9)	0.9 [0.7–1.2]	0.78	85 (47.8)	1.1 [0.7–1.5]	0.60
C/C	183 (14.5)	74 (15.2)	1.0 [0.7–1.4]	0.74	51 (16.5)	1.1 [0.7–1.6]	0.50	23 (12.9)	0.9 [0.5–1.5]	0.80
T/C + C/C	746 (59.3)	291 (59.9)	1.0 [0.8–1.2]	0.87	183 (59.4)	1.0 [0.7–1.2]	1.00	108 (60.7)	1.0 [0.7–1.4]	0.74
Allele T	1587 (63.1)	607 (62.4)	(ref.)	–	382 (62.0)	(ref.)	–	225 (63.2)	(ref.)	–
Allele C	929 (36.9)	365 (37.6)	1.0 [0.8–1.1]	0.76	234 (38.0)	1.0 [0.8–1.2]	0.65	131 (36.8)	0.9 [0.7–1.2]	0.99
rs1061651 (*TBX3*)										
T/T	414 (32.9)	154 (31.7)	(ref.)	–	98 (31.8)	(ref.)	-	56 (31.5)	(ref.)	–
T/C	596 (47.4)	240 (49.4)	1.0 [0.8–1.3]	0.54	153 (49.7)	1.0 [0.8–1.4]	0.61	87 (48.9)	1.0 [0.7–1.5]	0.71
C/C	248 (19.7)	92 (18.9)	0.9 [0.7–1.3]	1.00	57 (18.5)	0.9 [0.6–1.3]	0.92	35 (19.7)	1.0 [0.6–1.6]	0.90
T/C + C/C	844 (67.1)	332 (68.3)	1.0 [0.8–1.3]	0.64	210 (68.2)	1.0 [0.8–1.3]	0.73	122 (68.5)	1.0 [0.7–1.4]	0.73
Allele T	1424 (56.6)	548 (56.4)	(ref.)	–	349 (56.7)	(ref.)	–	199 (55.9)	(ref.)	–
Allele C	1092 (43.4)	424 (43.6)	1.0 [0.8–1.1]	0.93	267 (43.3)	0.9 [0.8–1.1]	0.98	157 (44.1)	1.0 [0.8–1.2]	0.84

*BC* breast cancer, *OC* ovarian cancer, *OR* odds ratio, *CI* confidence interval, Ref reference

^a^Fisher’s exact test; bold values are statistically significant (*p* < 0.05)

The single-locus analysis showed no significant differences between cases and controls in terms of genotype or allele distribution for rs4685:C > T, rs8853:T > C, or rs1061651:T > C, for the whole case group or either subgroup (*p* > 0.05) (Table 1).

However, the genotype and allele distribution for rs12366395:A > G (located in the *TBX3* gene) was significantly different for controls vs. the whole sample of *BRCA1/2*-negative cases and vs. subgroup A (*p* < 0.05) (Table 1). The minor allele frequency (MAF) (allele G) was higher in the whole sample (14.2%) and in subgroup A (15.3%) than in controls (11.4%) (OR = 1.2 [95% CI 1.0–1.6] *p* = 0.02; OR = 1.4 [95% CI 1.0–1.8] *p* = 0.009, respectively). Furthermore, we observed a significantly-increased BC risk for heterozygous individuals (A/G) and allele G carriers (A/G + G/G) in the whole sample (OR = 1.3 [95% CI 1.0–1.7] *p* = 0.01; OR = 1.3 [95% CI 1.0–1.7] *p* = 0.01, respectively). BC risk was also significantly higher in cases with genotype A/G and in allele G carriers from subgroup A (OR = 1.4 [95% CI 1.0–1.9] *p* = 0.01 and OR = 1.4 [95% CI 1.1–1.9] *p* = 0.008, respectively). We also analyzed the relationship between rs12366395:A > G and BC risk according to number of BC and/or OC cases per family (Table 2). No association between rs12366395:A > G and BC risk was found in cases from families with two BC/OC cases. However, BC risk was significantly higher in cases with three or more family members affected by BC and/or OC. In these families, the G allele frequency was 16.2% in BC cases vs. 11.4% in controls (OR = 1.5 [95% CI 1.0–2.1] *p* = 0.02), and both heterozygous individuals and allele G carriers had a significantly increased BC risk (OR = 1.5 [95% CI 1.0–2.2] *p* = 0.04 and OR = 1.5 [95% CI 1.0–2.2] *p* = 0.02, respectively) (Table 2). These results suggest that the allele G and allele G carrier genotypes are associated with risk in the context of a strong family history of BC. No association was found between rs12366395 and non-familial early-onset BC (≤ 50 years) (Table 1).

**Table 2:** Genotype and allele frequencies of rs4685 (*SF3B1*), rs12366395 (*TBX3*), rs72758040 (*MAP3K1*), rs8853 (*TBX3*), and rs1061651 (*TBX3*) according to number of BC cases per family in *BRCA1/2*-negative breast cancer cases and controls

		Families with 2 BC and/or OC cases (n = 163)			Families with ≥ 3 BC and/or OC cases (n = 145)		
Genotype or allele	Controls (%) (n = 1258)	BC cases (%)	OR [95% CI]	*p*-value^a^	BC cases (%)	OR [95% CI]	*p*-value^a^
rs4685 (*SF3B1)*							
C/C	504 (40.1)	75 (46.0)	(Ref.)	–	58 (40.0)	(Ref.)	–
C/T	560 (44.5)	70 (42.9)	0.8 [0.5–1.1]	0.33	69 (47.6)	1.0 [0.7–1.5]	0.77
T/T	194 (15.4)	18 (11.0)	0.6 [0.3–1.0]	0.10	18 (12.4)	0.8 [0.4–1.4]	0.50
C/T + T/T	754 (59.9)	88 (53.9)	0.7 [0.5–1.0]	0.15	87 (60.0)	1.0 [0.7–1.4]	1.00
Allele C	1568 (62.3)	220 (67.5)	(Ref.)	–	185 (63.8)	(Ref.)	–
Allele T	948 (37.7)	106 (32.5)	0.7 [0.6–1.0]	0.07	105 (36.2)	0.9 [0.7–1.2]	0.67
rs12366395 (*TBX3*)							
A/A	989 (78.6)	118 (72.4)	(Ref.)	–	102 (70.3)	(Ref.)	–
A/G	252 (20.0)	43 (26.4)	1.4 [0.9–2.0]	0.06	**39 (26.9)**	**1.5 [1.0–2.2]**	**0.04**
G/G	17 (1.4)	2 (1.2)	0.9 [0.2–4.3]	1.0	4 (2.8)	2.2 [0.7–6.9]	0.1
A/G + G/G	269 (21.4)	45 (27.6)	1.4 [0.9–2.0]	0.08	43 (29.7)	**1.5 [1.0–2.2]**	**0.02**
Allele A	2230 (88.6)	279 (85.6)	(Ref.)	–	243 (83.8)	(Ref.)	–
Allele G	286(11.4)	47 (14.4)	1.3 [0.9–1.8]	0.1	**47 (16.2)**	**1.5 [1.0–2.1]**	**0.02**
rs72758040 (*MAP3K1)*							
G/G	866 (68.8)	103 (63.4)	(Ref.)	–	92 (63.2)	(Ref.)	–
G/C	307 (24.4)	41 (25.0)	1.1 [0.7–1.6]	0.54	35 (24.3)	1.0 [0.7–1.6]	0.74
C/C	85 (6.8)	**19 (11.6)**	**1.8 [1.0–3.1]**	**0.03**	**18 (12.5)**	**1.9 [1.1–3.4]**	**0.01**
G/C + C/C	392 (31.2)	60 (36.6)	1.2 [0.9–1.8]	0.15	53 (36.8)	1.2 [0.8–1.8]	0.18
Allele G	2039 (81.0)	247 (75.9)	(Ref.)	–	219 (75.5)	(Ref.)	–
Allele C	477 (19.0)	**79 (24.1)**	**1.3 [1.0–1.7]**	**0.02**	**71 (24.5)**	**1.3 [1.0–1.8]**	**0.03**
rs8853 *(TBX3)*							
T/T	512 (40.7)	65 (39.9)	(Ref.)	–	60 (41.4)	(Ref.)	–
T/C	563 (44.8)	70 (42.9)	0.9 [0.6–1.4]	0.92	63 (43.4)	0.9 [0.6–1.3]	0.84
C/C	183 (14.5)	28 (17.2)	1.2 [0.7–1.9]	0.45	22 (15.2)	1.0 [0.6–1.7]	0.89
T/C + C/C	746 (59.3)	98 (60.1)	1.0 [0.7–1.4]	0.86	84 (58.6)	0.9 [0.6–1.3]	0.96
Allele T	1587 (63.1)	200 (61.3)	(Ref.)	–	183 (63.1)	(Ref.)	–
Allele C	929 (36.9)	126 (38.7)	1.0 [0.8–1.3]	0.58	107 (36.9)	0.9 [0.7–1.2]	0.95
rs1061651 (*TBX3*)							
T/T	414 (32.9)	52 (31.9)	(Ref.)	–	46 (31.7)	(Ref.)	–
T/C	596 (47.4)	77 (47.2)	1.0 [0.7–1.4]	0.92	76 (52.4)	1.1 [0.7–1.6]	0.49
C/C	248 (19.7)	34 (20.9)	1.0 [0.6–1.7]	0.72	23 (15.9)	0.8 [0.4–1.4]	0.60
T/C + C/C	844 (67.1)	111 (68.1)	1.0 [0.7–1.4]	0.85	99 (68.3)	1.0 [0.7–1.5]	0.85
Allele T	1424 (56.6)	181 (55.5)	(Ref.)	–	168 (57.9)	(Ref.)	–
Allele C	1092 (43.4)	145 (44.5)	1.0 [0.8–1.3]	0.75	122 (42.1)	0.9 [0.7–1.2]	0.71

*BC* breast cancer, *OC* ovarian cancer, *OR* odds ratio, *CI* confidence interval, *Ref* reference

^a^Fisher’s exact test; bold values are statistically significant (*p* < 0.05)

Similarly, the rs72758040:G > C (*MAP3K1* gene) genotype and allele distribution differed significantly between controls and the whole group of cases and between controls and subgroup A (*p* < 0.05) (Table 1). The MAF, allele C, was significantly higher in the whole sample (22.3%) and in cases with two or more family members with BC and/or OC (24.4%) vs. controls (19.0%) (OR = 1.2 [95% 1.0–1.4] *p* = 0.02; OR = 1.3 [95% 1.0–1.6] *p* = 0.003, respectively). This result indicates that the C allele is associated with increased BC risk. We also observed increased BC risk for homozygous C/C individuals in the whole sample and subgroup A cases (OR = 1.6 [95% CI 1.1–2.3] *p* = 0.01; OR = 1.9 [95% CI 1.3–3.0] *p* = 0.001, respectively). We then assessed the effect of rs72758040-C according to number of BC and/or OC cases per family (Table 2). The MAF (allele C) was significantly higher in families with two BC/OC cases (24.1%) and with three or more cases (24.5%) than controls (19.0%) (Table 2). Furthermore, BC risk was significantly higher in homozygous C/C individuals, both in the families with two BC/OC cases and with three or more cases (OR = 1.8 [CI 1.0–3.1] *p* = 0.03; OR = 1.9 [CI 1.1–3.4] *p* = 0.01, respectively). No association was observed between rs72758040 and cases from subgroup B. These results suggest that the C allele and C/C genotype are associated with elevated BC risk in cases with a family history of BC (Table 1).

### *Combined effect of* TBX3 *rs12366395-G and* MAP3K1 *rs72758040-C alleles on breast cancer risk*

As noted, *TBX3* and *MAP3K1* are driver or potential driver genes. Because the results indicated that rs12366395-G and rs72758040-C are associated with BC risk, we evaluated the combined effect of the two SNPs. Cases were divided into five groups for this analysis, according to risk allele count: zero (A/A + G/G), one (A/A + G/C, A/G + G/G), two (A/A + C/C, G/G + G/G, A/G + G/C), three (A/G + C/C, G/G + G/C), or four (G/G + C/C). Table 3 shows that the combined genotype distribution differed significantly in controls vs. the whole BC sample and in controls vs. subgroup A (global *p* = 0.009 and 0.0002, respectively), and BC risk increased in a dose-manner with number of risk alleles in the whole case group and subgroup A (*p*-trend = 0.01 and 0.001, respectively). No additive effect was observed in the early-onset BC group (diagnosis ≤ 50 year of age). We also analyzed this additive effect according number of BC and/or OC cases per family (Table 4). BC risk was elevated in the families with two BC and/or OC cases as well as in the families with the strongest history of BC (*p*-trend 0.05 and 0.003, respectively), These results indicate an additive effect of *TBX3* rs12366395 and *MAP3K1* rs72758040 on the risk conferred.

**Table 3:** Combined effect of rs12366395 (*TBX3*) and rs72758040 (*MAP3K1*) on breast cancer risk

Number of risk alleles^(a)^	Controls (n = 1258) (%)	All BC cases (n = 486)			Families with ≥ 2 BC and/or OC cases (n = 308)			Families with a single case, diagnosis at ≤ 50 years of age (n = 178)		
		BC cases (%)	OR [95% CI]	*p*-value value^b^	BC cases (%)	OR [95% CI]	*p*-value^b^	BC cases (%)	OR [95% CI]	*p*-value^b^
0 risk alleles	664 (52.8)	238 (49.0)	1.0 (Ref.)	–	145 (47.1)	1.0 (Ref.)	–	93 (52.2)	1.0 (Ref.)	–
1 risk allele	442 (35.1)	162 (33.3)	1.0 [0.8–1.2]	0.85	100 (32.5)	1.0 [0.7–1.3]	0.82	62 (34.8)	1.0 [0.7–1.4]	1.00
2 risk alleles	132 (10.5)	76 (15.6)	**1.6 [1.1–2.2]**	**0.004**	56 (18.2)	**1.9 [1.3–2.7]**	**0.0006**	20 (11.2)	1.0 [0.6–1.8]	0.78
3 risk alleles	19 (1.5)	7 (1.4)	1.0 [0.4–2.4]	1.00	4 (1.3)	1.0 [0.3–2.8]	1.00	3 (1.7)	1.1 [0.3–3.8]	0.74
4 risk alleles	1 (0.1)	3 (0.6)	**8.3 [0.8–80.9]**	**0.05**	3 (1.0)	**13.7 [0.4–133.1]**	**0.02**	0 (0.0)	2.3 [0.09–58.6]	1.00
*p*-trend^**(c)**^				**0.01**			**0.001**			0.81
Global *p* ^**(d)**^				**0.009**			**0.0002**			0.99

(a) 0 risk alleles: A/A + G/G; 1 risk allele: A/A + G/C, A/G + G/G; 2 risk alleles: A/A + C/C, G/G + G/G, A/G + G/C; 3 risk alleles: A/G + C/C, G/G + A/C; 4 risk alleles: G/G + C/C.; (b) Fisher’s exact test; (c) Chi-test for trend; (d) Chi-squared test for independence

*BC* breast cancer, *OC* ovarian cancer, *OR* odds ratio, *CI* confidence interval, *Ref* reference

Bold values are statistically significant (*p* < 0.05)

**Table 4:** Combined effect of rs12366395 (*TBX3*) and rs72758040 (*MAP3K1*) on breast cancer risk according to number of BC cases per family

Number of risk alleles^a^	Controls (n = 1258) (%)	Families with 2 BC and/or OC Cases (n = 163)			Families with ≥ 3 BC and/or OC Cases (n = 145)		
		BC Cases (%)	OR [95% CI]	*p*-value^b^	BC Cases (%)	OR [95% CI]	*p*-value^b^
0 Risk alleles	664 (52.8)	78 (47.9)	1.0 (Ref.)	–	67 (46.2)	1.0 (Ref.)	–
1 Risk allele	442 (35.1)	53 (32.5)	1.0 [0.7–1.4]	0.92	47 (32.4)	1.0 [0.7–1.4]	0.84
2 Risk alleles	132 (10.5)	**31 (19.0)**	**1.9 [1.2–3.1]**	**0.004**	**25 (17.2)**	**1.8 [1.1–3.0]**	**0.01**
3 Risk alleles	19 (1.5)	0 (0.0)	0.2 [0.01–3.6]	0.24	4 (2.8)	2.0 [0.6–6.3]	0.26
4 Risk alleles	1 (0.1)	1 (0.6)	8.5 [0.5–137.5]	0.20	2 (1.4)	**19.8 [1.7–221.6]**	**0.02**
p-trend^c^				**0.05**			**0.003**
Global p^d^				**0.003**			**0.001**

(a) 0 risk alleles: A/A + G/G; 1 risk allele: A/A + G/C, A/G + G/G; 2 risk alleles: A/A + C/C, G/G + G/G, A/G + G/C; 3 risk alleles: A/G + C/C, G/G + A/C; 4 risk alleles: G/G + C/C.; (b) Fisher’s exact test; (c) Chi-test for trend; (d) Chi-squared test for independence

*BC* breast cancer, *OC* ovarian cancer, *OR* odds ratios, *CI* confidence interval, *Ref* reference

Bold values are statistically significant (*p* < 0.05)

## Discussion

Cancer is essentially a disease of the genome, and a large number of somatic mutations accumulate during the process of tumorigenesis. Some of those mutations contribute to tumor initiation/progression and are known as driver mutations [2]. The driver mutations and mutational processes underlying BC have not been comprehensively explored [6].

The T-box transcription factor 3 gene (*TBX3*) is a member of a gene family that shares a common DNA-binding domain, the T-box. T-box genes encode a transcription factor that regulates stem cell pluripotency-associated and reprogramming factors and is involved in normal breast development [18, 19]. Furthermore, *TBX3* overexpression has been observed in primary breast tumors and BC cell lines with elevated expression in estrogen receptor-positive tumor cells [20]. Recently, somatic variations in *TBX3* have been classified as BC driver mutations [6–9, 21, 22]. Göhler et al. [10] studied the rs12366395 germline variation in a Swedish cohort, reporting that minor allele carriers had decreased BC risk (OR = 0.83 [CI 95% 0.69–1.00] dominant model). In contrast, our results show that the rs12366395-G allele is associated with increased risk in familial BC. Various authors support the hypothesis that genetic factors differ by race and ethnicity as they relate to BC [23]. Today’s Chilean population stems from the admixture of Amerindian peoples with sixteenth- and seventeenth-century Spanish settlers. Nineteenth-century migrations of Germans, Italians, Arabs, and Croatians have had only a minor impact on the overall population (accounting for less than 4% of total inhabitants) and are restricted to the specific locations of the country where they settled [24]. The relationship between ethnicity, Amerindian admixture, genetic markers, and socioeconomic strata has been extensively studied in Chile [25–27]. Therefore, any discrepancies between Göhler et al. [10] and the present work might be explained by the different genetic backgrounds of the samples. To date, no information on *TBX3* rs12366395 has been reported for any other population in the world. *TBX3* has no known function in adult tissues but is frequently overexpressed in a wide range of epithelial and mesenchymal-derived cancers. This overexpression greatly impacts several hallmarks of cancer, promoting proliferation, tumor formation, angiogenesis, invasion, and metastasis [15]. rs12366395 is located in the *TBX3* 5’UTR region. Changes in this regulatory region could alter the secondary structure of the 5’UTR region and affect translation speed. On the other hand, the *TBX3* 5’UTR region extends for 975 nt rs12366395 is found at nt 778 of the 5'UTR region, and this SNP is 198 nt upstream from the AUG start codon (genomic locations mentioned refer to TBX3 transcript variant 1 ENST00000349155.7). The functional consequences of sequence variants within the mRNA 5′ leader (i.e., the region upstream of the initiator codon) may impact translation output [28]. For example, while assessing for oncogenic changes associated with prostate cancer, Wang et al. (2009) [29], identified a G-to-A somatic mutation that mapped within the δ-catenin 5′ leader region, nine nucleotides upstream of the AUG codon. The presence of the A allele in reporter mRNAs resulted in a three- to seven-fold increase in protein expression relative to mRNAs harboring the G allele, with no effect on mRNA levels. Therefore, given the location of rs12366395 in the *TBX3* 5’UTR, this SNP could produce and increase TBX3 protein levels in cells, which could explain the effect on BC risk.

The SNP rs72758040 is located in the promotor region of the *MAP3K1* gene at 439 nt upstream from Transcription Start Site (ENST00000399503.4) [10]. There is evidence to suggest that *MAP3K1* is a potential driver gene in BC and acts within the MAP-signaling pathway, which triggers the expression of genes crucial for angiogenesis, proliferation, and cell migration [6]. Thus, it is important to determine whether the SNP rs72758040 contributes to HBC risk in the Chilean population. In this study, we found that rs72758040 was significantly associated with familial BC risk in a Caucasian-Amerindian South American population. These results are in agreement with those published by Göhler et al. in a Swedish sporadic BC cohort. Both studies observed an increased BC risk for homozygous C/C individuals. Nevertheless, there are no other publications in the literature on *MAP3K1* rs72758040 and BC. Therefore, the results should be replicated in other populations to clarify the role on this SNP in risk. Moreover, as *MAP3K1* seems to be a potential driver gene, functional studies would be helpful, in order to confirm that this SNP is a BC driver variation. One important issue to consider is that the genotype distribution of rs72758040 in *MAP3K1* gene is in a Hardy–Weinberg disequilibrium, which could distort the results. The possibility that different selective factors may directly or indirectly alter the association between rs72758040 and BC risk cannot be discarded.

**Fig. 1:**
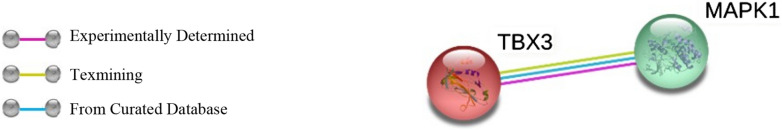
Protein association network according to STRING analysis, showing interactions of TBX3 with MAPK1. Line colors indicate the different validations of the interaction between the two proteins

As our results showed that SNPs rs12366395 (*TBX3*) and rs72758040 (*MAP3K1*) were associated with BC risk, we evaluated their combined effect and constructed a genetic score based on risk allele count. A dose–response association was observed for familial BC (Table 3). As noted above, *TBX3* is a transcription factor frequently overexpressed in various types of human cancers, including BC [10], while the *MAP3K1* gene induces MAP-kinase pathway. There is no information in the literature regarding the interaction between these two genes. To assess for an interaction between TBX3 and MAP3K1 proteins that could explain a synergistic effect on risk, we used the default parameters of the STRING software v11.0 (https://string-db.org/) to analyze the TBX3-MAP3K1 interaction. We found that TBX3 related directly to MAPK1 (Fig. 1), which is a protein that interact directly with MAP3K1 in MAP signaling. Further studies are necessary to evaluate the functional impact of rs12366395-G (*TBX3*) and rs72758040-C (*MAP3K1*) on BC tumorigenesis. Although our study provides evidence for an association of rs12366395 and rs72758040 with BC risk, certain limitations must be considered. Firstly, the genotype distribution of rs72758040 did not conform to the Hardy–Weinberg expectations, which may distort the results. Secondly, the sample size of the whole group in the present study is sufficient to yield 80% power; nevertheless, the sample size limits the subgroup analyses. Therefore, these results should be replicated using subgroups with larger sample sizes.

## Conclusion

Our study suggests that germline variants in driver genes *TBX3* (rs12366395) and *MAP3K1* (rs72758040) may influence BC risk in *BRCA1/2*-negative Chilean families. Moreover, the presence of rs12366395-G and rs72758040-C could increase BC risk in a Chilean population. Given that this is the first association study of these SNPs in a South American population, analyses in other populations would be helpful to clarify their role in BC tumorigenesis. Furthermore, functional studies should be performed to determine the biological impact of these mutations.

## Materials and methods

### Families

We reviewed records from the Servicio de Salud del Area Metropolitana de Santiago, Corporación Nacional del Cáncer (CONAC) and private providers in Santiago to identify BC patients from high-risk *BRCA1/2*-negative Chilean families. A total of 486 women with BC were enrolled (one case per family). We tested index cases for *BRCA1* and *BRCA2* mutations as previously described [16], then developed pedigrees based on the index case with the greatest probability of carrying a deleterious mutation. None of the families met strict criteria for BC-related syndromes such as Li-Fraumeni, ataxia-telangiectasia, or Cowden disease.

We performed extensive ancestry interviews with several family members of each case, including persons from different generations. All families self-reported exclusive Chilean ancestry for multiple generations. Table 5 shows the specific characteristics of the families selected according to the inclusion criteria. A total of 18.1% (88/486) of the study families had cases of bilateral BC; 9.7% (47/486) had cases of both BC and ovarian cancer (OC); and 1.1% (5/486) had cases of male BC. The mean age at diagnosis was 44.3 years, with 78.4% cases diagnosed before 50 years of age.

**Table 5:** Inclusion criteria for study families

Inclusion criteria	Families: n (%)
Three or more family members with breast and/or ovarian cancer	145 (29.8%)
Two family members with breast and/or ovarian cancer	163 (33.6%)
Single affected individual with breast cancer ≤ age 35	87 (17.9%)
Single affected individual with breast cancer age 36–50	91 (18.7%)
Total	486 (100%)

This study was approved by the Institutional Review Board of the University of Chile School of Medicine (Grant Number 1200049, March 2020). Written informed consent was obtained from all participants. All methods were performed in accordance with the relevant guidelines and regulations.

### Control population

CONAC files were also reviewed to recruit healthy women (control group n = 1258). Controls were unrelated to the study families and reported no personal or family history of cancer. All controls confirmed that they were of Chilean ancestry, and over 90% were residents of Santiago. Case and control groups were matched for age and socioeconomic status. Participants provided written informed consent, and DNA samples were obtained in accordance with all ethical and legal requirements.

### Genotyping analysis

Genomic DNA was extracted from peripheral blood lymphocytes of the 486 cases from the selected high-risk families and 1258 controls, following Chomczynski and Sacchi [17].

Genotyping of the SNPs rs4685:C > T, rs12366395:A > G, rs72758040:G > C, rs8853:C > T, and rs1061651:T > C was performed using the commercially-available TaqMan Genotyping Assay (Applied Biosystems, Foster City, CA) (assay IDs C___2834688_20, C__25624196_10, C__97102043_10, C___1412080_10, and C___1412077_20 respectively). The reaction was carried out as described by Fernandez-Moya et al. [12].

### Statistical analysis

The Hardy–Weinberg equilibrium assumption was assessed in the control sample using a goodness-of-fit chi-square test (HW Chisq function, “Hardy Weinberg” package v1.4.1). Fisher's exact test was used to test the association between genotypes and/or alleles for cases and controls. Odds ratios (OR) with 95% confidence intervals (CI) were calculated to estimate the strength of the associations in cases and controls. For all analyses, the level of significance was set at p- ≤ 0.05. GraphPad Prism software v6.0 for Windows 10, CA, USA, www.graphpad.com) was used for the Fisher's exact test and odds ratio analyses. A chi-square test for trend was performed identify any additive effects of the SNPs (‘p-trend’ was determined using the Stata/MP v13.0 for Windows 10, Unix-StataCorp, College Station, TX, USA; ‘p-trend’ package).

### Methodology authority statement

All methods using in this study can be found in a previous published article of our authority [12].

## Declarations

### Availability of data and materials

All data are shown within the manuscript.
